# Infographic: vitrectomy plus encircling band vs. vitrectomy alone for the treatment of pseudophakic retinal detachment (VIPER) study

**DOI:** 10.1038/s41433-021-01529-7

**Published:** 2021-05-26

**Authors:** Alexander Mehta, Salman Sadiq, Nikolaos Tzoumas, Anna Song, Declan Murphy, Islam Mostafa, Ali Ghareeb, Mohaimen Al-Zubaidy, David Steel

**Affiliations:** Bioscience Institute, Newcastle University, Newcastle Upon Tyne, UK

**Keywords:** Outcomes research, Anatomy

**Reference to original study**: Walter P, Hellmich M, Baumgarten S, Schiller P, Limburg E, Agostini H et al. Vitrectomy with and without encircling band for pseudophakic retinal detachment: VIPER Study Report No 2-main results. Br J Ophthalmol 2017; 101(6):712-718.

**Fig. 1:**
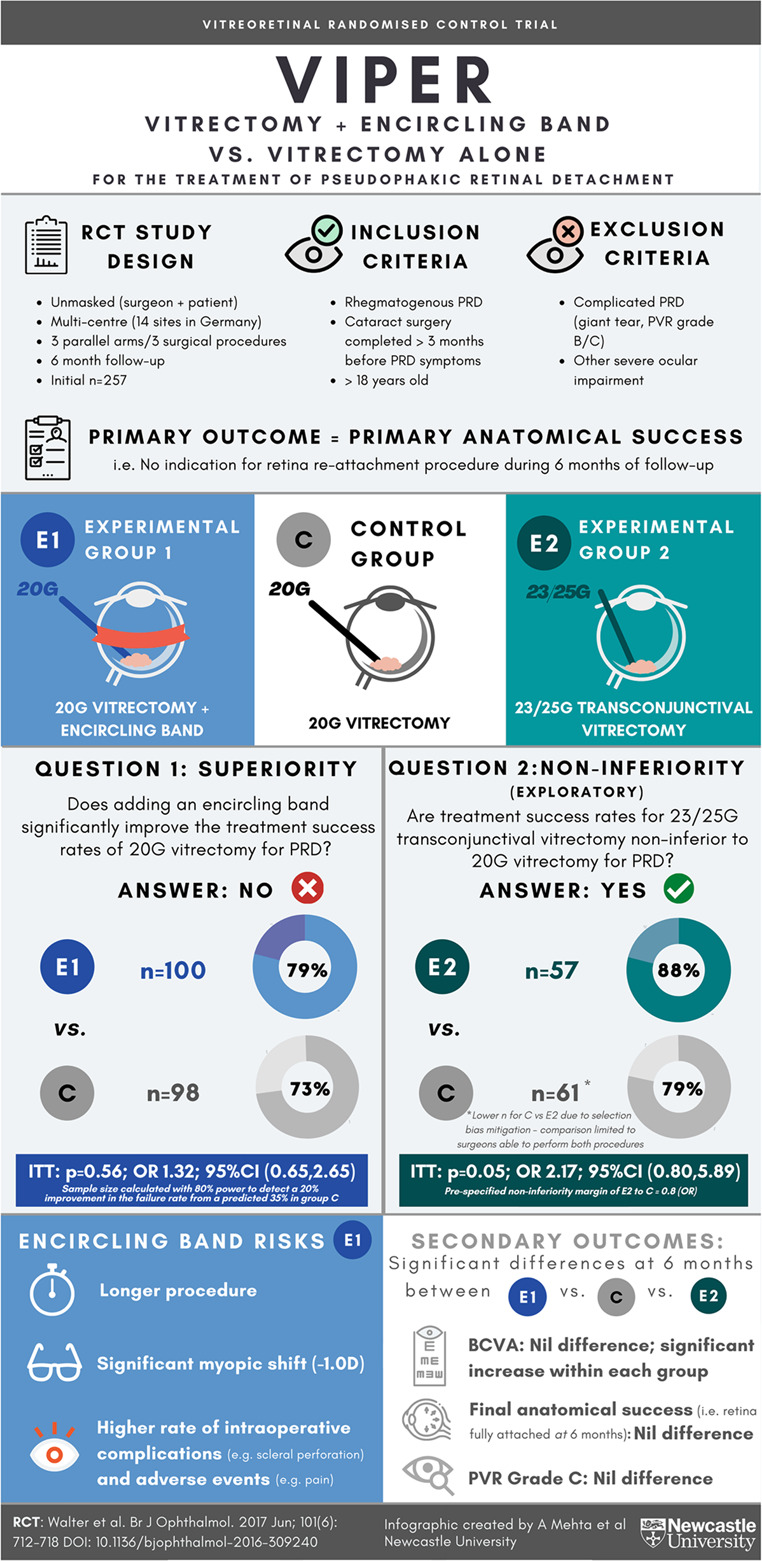
The Vitrectomy Plus Encircling Band vs. Vitrectomy Alone for the Treatment of Pseudophakic Retinal Detachment (VIPER) randomised control trial showed that performing scleral buckling in addition to vitrectomy does not significantly reduce the risk of follow-up retinal reattachment surgery. For management with vitrectomy alone, small gauge transconjunctival vitrectomy (23/25 G) is not inferior to the conventional 20 G technique. RCT randomised control trial, PRD pseudophakic retinal detachment, ITT intention to treat, OR odds ratio, CI confidence interval, BCVA best corrected visual acuity, PVR proliferative vitreoretinopathy.

